# Prevalence of psychiatric diagnosis and related psychopathological symptoms among patients with COVID-19 during the second wave of the pandemic

**DOI:** 10.1186/s12992-021-00694-4

**Published:** 2021-04-08

**Authors:** Zhiyang Zhang, Yi Feng, Rui Song, Di Yang, Xuefei Duan

**Affiliations:** 1 The National Clinical Research Center for Mental Disorders & Beijing Key Laboratory of Mental Disorders & Beijing institute for Brain Disorders Center of Schizophrenia, Beijing Anding Hospital, Capital Medical University, Beijing, China; 2 Advanced Innovation Center for Human Brain Protection, Capital Medical University, Beijing, China; 3 Mental Health Center, Central University of Finance and Economics, 39 South College Road, Haidian District, Beijing, 100081 China; 4 Faculty of Psychology, Beijing Normal University, Beijing, China; 5 Center of Infectious Diseases, Beijing Ditan Hospital, Capital Medical University, Beijing, China; 6 Department of General Medicine, Beijing Ditan Hospital, Capital Medical University, No. 8 Jingshun East Street, Chaoyang District, Beijing, 100015 China

**Keywords:** Mental health, Psychopathological symptoms, Infected patients, Risk factors, COVID-19

## Abstract

**Background:**

The possibility of psychopathological symptoms and related risk factors among normal persons and patients infected during the outbreak of COVID-19 has been widely investigated. The mental health outcomes of the second wave of the pandemic remain unclear, especially those of patients with an infection. Thus, this study aims to explore the prevalence of and related risk factors associated with psychopathological symptoms among patients infected with COVID-19 during the second wave.

**Method:**

A cross-sectional survey was conducted in five isolated wards of a designated hospital in Beijing, China, from July 1 to July 15, 2020. The Mini International Neuropsychiatric Interview (MINI) was conducted to assess psychiatric disorders, and a series of scales were used to measure self-reported psychopathological symptoms and psychosomatic factors. Multivariate regression analysis was used to analyze the risk factors associated with psychopathological symptoms.

**Results:**

Among 119 participants with infections, the prevalence of generalized anxiety symptoms (51.3%), depressive symptoms (41.2%), and posttraumatic stress symptoms (PTSS)/posttraumatic stress disorder (PTSD) symptoms (33.6%) was observed. Loneliness, hope, coping strategies, and history of mental disorders were the shared risk or protective factors across several psychopathological symptoms. The perceived impact of COVID-19 is the specific risk factor associated with state anxiety symptoms.

**Conclusions:**

The prevalence of symptoms of depression, anxiety, and PTSS/PTSD is high among patients with infections during the second wave of the pandemic in Beijing. Clinical doctors must realize that these patients will probably experience depressive disorder, anxiety disorders, and PTSS/PTSD, as well as some neuropsychiatric syndromes. Specific mental health care is urgently required to help patients manage the virus during the second wave of the pandemic.

## Introduction

The first case of new coronavirus disease (COVID-19), the spread of which has resulted in a global pandemic, was reported in Wuhan City, Hubei Province, China, in December 2019 [1]. Patients suffering from pain also dealt with major mental health issues in the face of increasing number of infections and deaths. China immediately implemented emergency policies to manage the COVID-19 pandemic, such as early detection, isolation of patients with suspected and confirmed infections, and the establishment of isolation units and hospitals [2]. These policies quickly controlled the COVID-19 pandemic. The infected cases nationwide decreased to less than 20 per day, and there were none in Beijing from April 29 to June 10, 2020. The period from late 2019 to June 10 is regarded as the “first wave” of pandemic in this study.

However, from June 11 to July 15, 2020, the second wave of COVID-19 began, and in Beijing, China, the infected cases increased from 0 to 335. After many countries had controlled the first wave of the pandemic, they relaxed their containment and physical distancing control measures, causing the infection rate to increase accordingly [3, 4]. This worldwide resurgence of COVID-19 cases indicated that the second wave of the pandemic was occurring [5–7]. However, few studies have explored the effect of the second wave of the pandemic on mental health. Thus, investigating the mental health status of the public and special groups during the second wave of the pandemic is necessary.

The general medical complications of COVID-19 have attracted substantial attention, but the possible direct impact of this pandemic on mental conditions and neuronophagia has rarely been studied [8]. China’s treatment guidelines stipulate that patients with COVID-19 infections should be treated in isolated hospitals. Autism, anger, anxiety, depressive disorder, insomnia, and posttraumatic stress symptoms (PTSS)/posttraumatic stress disorder (PTSD) have been observed in patients [9–11]. Thus, we assume that the causes of these problems might be social isolation, the surrounding risks and uncertainties, pain and suffering, drug reactions, worrying about spreading the contagion to others, and negative information consumed on social networks [12]. Studies have investigated the first wave of the pandemic and observed mental health problems such as PTSS/PTSD, anxiety, and depressive symptoms among health care workers or the general population [13–16]. Few studies have investigated mental health problems among patients infected by COVID-19 during the second wave.

Our study aimed to explore the prevalence of psychopathological symptoms and related risk factors among patients infected by COVID-19 during the second wave in Beijing, China. To ensure that our assessment of the prevalence of psychopathological symptoms was more credible than those in previous studies, we used a mixed method, i.e., psychiatric diagnosis by experienced psychiatrists and self-report measures by participants. In addition to sociodemographic characteristics and COVID-19-related factors, we used mental-health-related factors from the perspective of psychological factors (i.e., loneliness, hope), behavior factors (i.e., coping strategies), and external factors (i.e., social support) as possible risk or protective factors for psychopathological symptoms.

## Methods

### Participants and study design

This study used a cross-sectional design and was conducted during the second wave of COVID-19, from July 1 to 15, 2020. The sample was patients with infections in Beijing Ditan Hospital, the designated isolation hospital for COVID-19 infections in Beijing, China. Cluster sampling was used to recruit the participants. We invited 180 inpatients from five isolated wards to participate in this study, which included completing the Mini International Neuropsychiatric Interview (MINI) and self-reported questionnaires.

On the day before each participant’s discharge from the hospital, two experienced clinical psychiatrists wearing protective clothing administered the MINI face to face. The standard MINI version was used to assess the 17 most common disorders related to mental health (e.g., PTSD, anxiety disorder). The patients were told that the review was structured and “yes” (indicating the patient was unlikely to have a major psychiatric disorder) or “no” (indicating the patient was likely to have a major psychiatric disorder) answers were required.

The self-report measures were distributed to the participants by sending them an electronic link to the questionnaires. All participants completed the questionnaires on cellphones. Each participant was informed of the purpose and procedures of this study before the survey and that they had the right to withdraw at any time during this study. Online informed consent was obtained from each participant before they completed the study.

Participants meeting the following criteria were included: (1) patients who were diagnosed with a COVID-19 infection, (2) Chinese citizens who understood Chinese, and (3) those who were infected during the second wave of the pandemic in Beijing. Of the 180 patients invited to participate in this study, 61 withdrew from the survey because they were unwilling to complete the interview or questionnaires. The final sample size was 119 and response rate was 66.1%. This study was approved by the Beijing Ditan Hospital Ethics Committee.

### Measures

#### Sociodemographic characteristics

Various scales and demographic data were collected, including sex, age, nationality, education, clinically diagnosed type of infection, community risk (announced by the Beijing Municipal Government during the second wave), annual income, and history of mental disorder (confirmed by two psychiatrists).

#### COVID-19-related factors

Participants were surveyed to indicate the frequency of exposure to information or news related to COVID-19. We self-designed two items: “How many times per day in the past 2 weeks have you browsed the information related to the pandemic?” and “How many hours per day in the past 2 weeks have you browsed pandemic-related information?” The scoring criteria for the first and second items were from “0 times” to “20 times” with a total score of 21 points and from “0 h” to “8 h” with a total score of 8 points, respectively. The responses formed the composite score of exposure to COVID-19-related information (Cronbach’s α = 0.52), with higher mean scores indicating a higher exposure to COVID-19-related information or news.

Participants were surveyed to determine the perceived impact of COVID-19. Four items were designed by the authors and used to measure the impact on economic income, daily life, work or study, and interpersonal relationships, with responses ranging from 1 (*totally not*) to 5 (*to a large extent*). We calculated a composite score of perceived impact (Cronbach’s α = 0.73) after different answers to these items from 1 (totally not) to 5 (to a large extent), with higher scores indicating a greater perceived impact of the COVID-19 pandemic.

#### Psychological factors

Loneliness and hope were assessed as two psychological factors associated with psychopathological symptoms. The 6-item short version of the De Jong Gierveld Loneliness Scale was used to assess loneliness [17]. For each item, participants were asked to indicate the extent to which corresponding situations had occurred (e.g., “I experience a general sense of emptiness,” “I miss having people around”) on a 5-point scale (1 = *never*, 5 = *always*), with validated Cronbach’s α = 0.70 ~ 0.76. We calculated a composite loneliness score (Cronbach’s α = 0.55), with higher scores indicating a higher level of loneliness.

Hope was assessed by the Hope Scale, comprising 12 items concerning feelings of hope, with validated Cronbach’s α = 0.74 ~ 0.84 [18]. The Hope Scale defined hope as the process of thinking about personal goals and the motivation to advance toward (agency subscale) and the ways to achieve (pathways scale) those goals (e.g., “I energetically pursue my goals,” “I can think of many ways to get out of a jam”). Responses were on a 7-point scale from 1 (*definitely false*) to 7 (*definitely true*). We calculated a composite hope score (Cronbach’s α = 0.91), with higher scores indicating a higher level of hope.

#### Coping strategies

The 15-item coping inventory (COPE) was used to assess the coping strategies that participants used to manage their stress [19, 20]. COPE comprises four subscales: active coping, avoidant coping, emotion-focused coping, and acceptance coping. Participants were asked to rate on a 7-point scale from 1 (*never*) to 7 (*always*) how frequently they used each coping strategy (e.g., “I concentrate my efforts on doing something about it,” “I pretend that it hasn’t really happened,” “I discuss my feelings with someone,” “I learn to live with it”). In this study, the composite cope subscale score was Cronbach’s α = 0.50 ~ 0.87, with higher scores indicating a higher frequency of using a coping strategy in the corresponding subscale.

#### Social supports

Social supports were assessed by the Multidimensional Scale of Perceived Social Support (MSPSS) [21], comprising 12 items. The MSPSS comprises three subscales: perceived support from family, from friends, and from a significant other. Examples of these items are “There is a special person who is around when I am in need” and “I can talk about my problems with my friends.” Each item was rated on a 7-point Likert scale, ranging from 1 (*very strongly disagree*) to 7 (*very strongly agree*). The composite social support was calculated (Cronbach’s α = 0.95), with higher scores indicating a higher level of perceived social support.

#### Psychopathological symptoms

We measured symptoms of generalized anxiety, state anxiety, depression, COVID-19-related PTSS/PTSD, somatization, interpersonal sensitivity, hostility, paranoid ideation, and psychoticism as psychopathological symptoms.

Generalized anxiety was assessed by the Generalized Anxiety Disorder Scale (GAD-7), a self-reported screening scale comprising seven items on a 4-point scale from 0 (*not at all*) to 3 (*nearly every day*), with a higher total score indicating severer anxiety symptoms [22]. The Chinese version of GAD-7 has been validated and demonstrated good reliability (Cronbach's α = 0.89) [23]. In our study, the cut-off score for anxiety symptoms was 5 [23], and internal consistency was excellent (Cronbach’s α = 0.94).

We also measured the state anxiety level of the patients while hospitalized. The State-Trait Anxiety Inventory-State (SASI-S) was used to screen for situation-related anxiety [24]. The SASI-S comprises 20 items on a 4-point scale from 1 (*not at all*) to 4 (*very much*), with a higher summative score indicating higher levels of state anxiety, and has demonstrated excellent internal consistency (Cronbach’s α = 0.94) [25]. In this study, the cut-off score for a state anxiety symptom was 41 [26] and the Cronbach’s α was 0.91.

Similar to GAD-7, the self-screen 9-item Patient Health Questionnaire (PHQ-9) was used to assess the frequency of the occurrence of depressive symptoms over the past two weeks on a 4-point Likert scale from 0 (*not at all*) to 3 (*nearly every day*) [27]. The PHQ-9 has been validated in China (Cronbach’s α = 0.86) [28], and we produced a summative score, with higher scores indicating severer depressive symptoms (Cronbach’s α = 0.91). The cut-off score for a depressive symptom was 5 in this study [27].

The impact of Events Scale-Revised (IES-R) was adapted to measure COVID-19-related PTSS/PTSD [29]. The IES-R comprises 22 items on a 5-point Likert-type scale (0 = *not at all*; 4 = *always*) to produce a summative score, with higher scores indicating a higher level of events-related PTSS/PTSD. Participants were asked to state the frequency with which each symptom had occurred in the past week, and the event refers to the COVID-19 event in this study. The IES-R has been validated in COVID-19 studies in China [30, 31]. We calculated a COVID-19-related PTSS/PTSD composite score (Cronbach’s α = 0.97) with the cut-off score being 24 [32].

The somatization subscale, interpersonal sensitivity subscale, hostility subscale, paranoid ideation subscale, and psychoticism subscale of the Brief Symptom Inventory (BSI) were used to assess the five specific psychopathological symptoms [33]. Respondents ranked each feeling item on a 5-point scale ranging from 1 (*not at all*) to 5 (*extremely*) in the past seven days, with higher scores indicating severer sub-dimensional symptoms. This study demonstrated the internal consistency of the five subscales was good (Cronbach’s α = 0.83 ~ 0.87). To our knowledge, few studies have provided BSI-53 subscale cut-off scores to diagnose specific psychiatric illness [34].

### Data analysis

We used both the MINI diagnostic outcome and the self-reported clinical symptoms outcome to calculate the prevalence of psychopathological symptoms among the participants. Descriptive statistics for sociodemographic and clinical characteristics were first conducted. Next, a chi-square test was used to compare the psychopathological symptoms between males and females. Finally, hierarchical linear regression models were used to explore the contribution of various factors to psychopathological symptoms. Based on our study aim and literature [30], our final order of different independents was determined. Sociodemographic characteristics were first entered to test their relationship with psychopathological symptoms in step 1, followed by COVID-19-related factors in step 2, psychological factors in step 3, cope strategies in step 4, and social support in model 5. We used the bias-corrected bootstrap method with 95% confidence intervals to test the regression models. In addition, the multicollinearity test demonstrated that all tolerance values were higher than 0.1 and that all variance inflation factor values were less than 10 for all variables entered in the models, indicating that the regression models were acceptable [35].

The analysis of the chi-square test and regression used self-reported symptoms as the dependent variables due to limited number of positive cases and dichotomous outcome variables diagnosed by MINI. All analyses were performed by using SPSS version 23.0 and R version 4.0.2. The statistical significance level was set at 0.05 (two-sided).

## Results

### Sociodemographic and clinical characteristics

The final sample comprised 119 participants. As shown in Table 1, the average age of the participants was 40.25 (*SD* = 11.50) years. The characteristics that represented a majority of the patients were as follows: male (62.2%), Han ethnic (93.3%), a lower education level (68.9%), and annual family income lower than 100,000 RMB (72.3%). Most patients were diagnosed as “Normal” infected (84%) and 98.3% did not have a history of mental disorders. According to the MINI diagnosis conducted by experienced clinical psychiatrists, 12.61% patients suffered from anxiety spectrum disorder, 5.9% suffered from depressive disorder, and 9.24% suffered from PTSS or PTSD. The diagnosis outcome of the prevalence of psychopathological symptoms on the basis of the self-reported questionnaires was higher than that by MINI: of the patients, 51.3% had generalized anxiety and state anxiety symptoms, 41.2% had depressive symptoms and 33.6% had PTSS/PTSD symptoms.

**Table 1:** Sociodemographic and clinical characteristics of the study sample (*N* = 119)

Variables	Number (***n***)	Percent (***%***)	
Mean age (*SD*)		40.25 ± 11.50	
Sex			
Male		74	62.2
Female		45	37.8
Ethnic group			
Han		111	93.3
Others		8	6.7
Education level			
Junior school or lower		82	68.9
High school		21	17.7
College or above		16	13.4
Annual family income			
< 30,000		31	26.1
30,000 ~ 60,000		33	27.7
70,000 ~ 100,000		22	18.5
100,000 ~ 150,000		16	13.4
> 150,000		17	14.3
Community risk			
No risk		2	1.7
Low risk		16	13.4
Moderate risk		44	37.0
High risk		57	47.9
Type of infection			
Asymptomatic		5	4.2
Light		14	11.8
Normal		100	84.0
Severe		0	0
History of mental disorder			
Yes		2	1.7
No		117	98.3
MINI diagnosis			
Anxiety spectrum disorder		15	12.61
Depressive disorder		7	5.9
PTSD		11	9.24
Manic episode		2	1.68
Obsessive-compulsive disorder		3	2.52
Self-reported Scale diagnosis			
Generalized anxiety symptoms		61	51.3
State anxiety symptoms		61	51.3
Depressive symptoms		49	41.2
PTSS/PTSD symptoms		40	33.6

*Note*. The unit of annual income is CNY yuan

### Prevalence of psychopathological symptoms

**Fig. 1:**
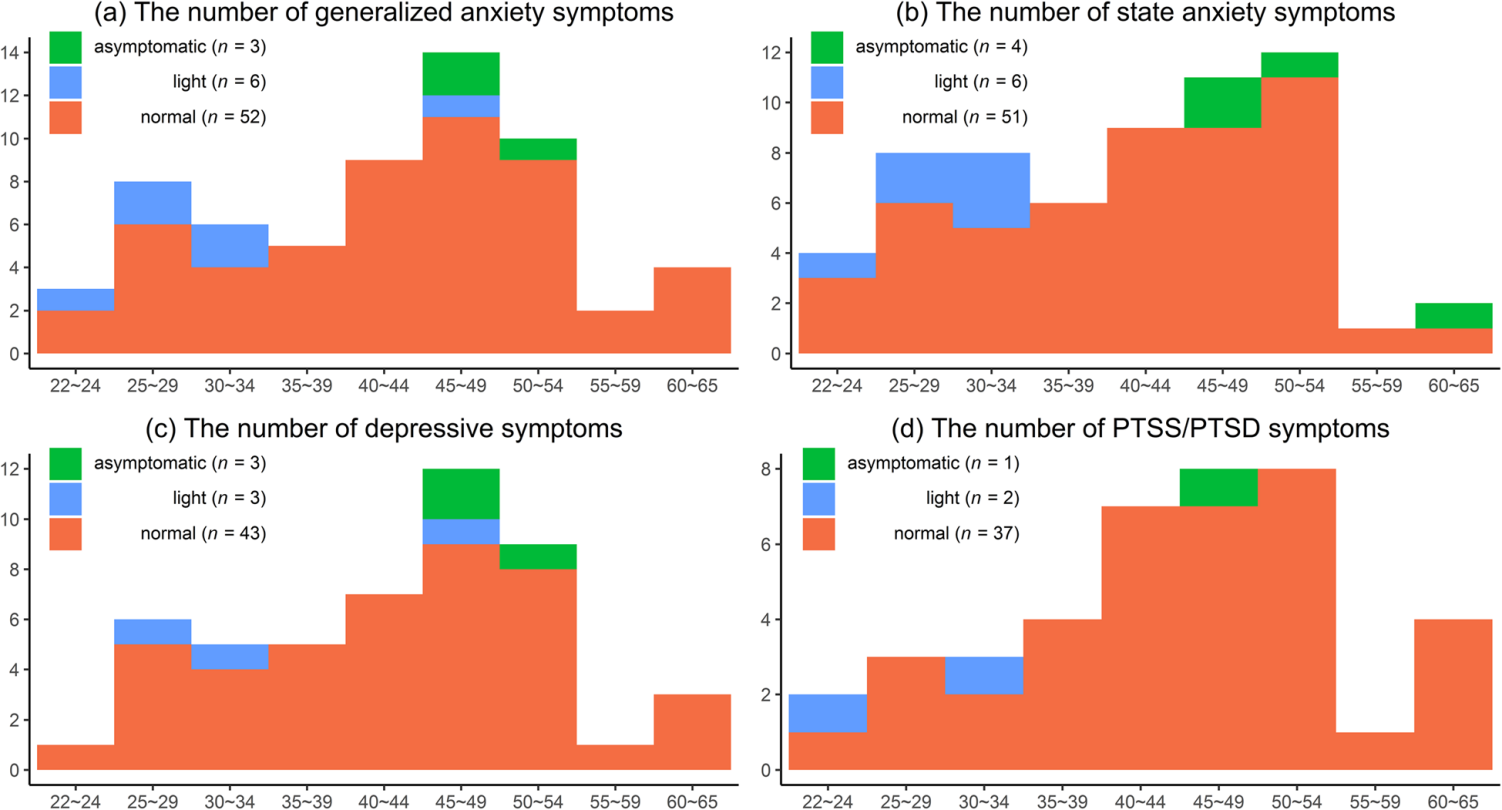
Prevalence of psychopathological symptoms among three types of infection. *Note*. The *n* indicates the number of positive cases of psychopathological symptoms (i.e., generalized anxiety, state anxiety, depression, PTSS/PTSD) in different age groups among three types of infection

Prevalence of self-reported psychopathological symptoms was high among patients. More than half had generalized anxiety (51.3%) or state anxiety (51.3%) symptoms. Nearly half of the patients had depressive symptoms (41.2%). As shown in Fig. 1, the patients with the normal infection type demonstrated a relatively higher proportion of having psychopathological symptoms than those with light and severe infection: 85.2% had generalized anxiety symptoms, 83.6% had state anxiety symptoms, 87.8% had depressive symptoms, and 92.5% had PTSS/PTSD symptoms. Patients aged 40 to 55 years accounted for a relatively higher proportion of psychopathological symptoms than the patients of other ages did. In addition, the chi-square test demonstrated no significant difference between males and females in generalized anxiety (*χ*^2^(1) = 2.21, *p* = .137), state anxiety (*χ*^2^(1) = 0.53, *p* = .465), or depressive symptoms (*χ*^2^(1) = 0.90, *p* = .343). However, female patients had a significantly higher proportion of PTSS/PTSD symptoms (*χ*^2^(1) = 5.53, *p* = .019) than male patients did.

### Associated factors with psychopathological symptoms

The results of the regression demonstrated that loneliness was the only shared risk factor across all psychopathological symptoms. As shown in Table 2, in addition to loneliness, hope was the shared risk factor across generalized anxiety (*β* = 0.23, *p* = .040), depression (*β* = 0.26, *p* = .018), PTSS/PTSD (*β* = 0.29, *p* = .009), paranoid ideation (*β* = 0.22, *p* = .017), and psychoticism symptoms (*β* = 0.18, *p* = .026). History of mental disorder was the shared risk factor across PTSS/PTSD (*β* = 0.25, *p* = .018), interpersonal sensitivity (*β* = 0.31, *p* = .002), hostility (*β* = 0.48, *p* < .001), paranoid ideation (*β* = 0.58, *p* < .001), and psychoticism (*β* = 0.62, *p* < .001). Avoidant coping strategy was the shared risk factor across generalized anxiety (*β* = 0.22, *p* = .024), depression (*β* = 0.26, *p* = .009), somatization (*β* = 0.25, *p* = .021), hostility (*β* = 0.20, *p* = .019), and psychoticism (*β* = 0.22, *p* = .003). Acceptance coping was the shared protective factor across generalized anxiety (*β* = − 0.43, *p* = .010) and psychoticism (*β* = − 0.30, *p* = .016). Support from significant others was the shared risk factor across state anxiety (*β* = 0.32, *p* = .050) and depressive symptoms (*β* = 0.37, *p* = .030). Age was the shared risk factors across generalized anxiety (*β* = 0.18, *p* = .038) and PTSS/PTSD symptoms (*β* = 0.23, *p* = .009). Being male was associated with fewer PTSS/PTSD (*β* = − 0.21, *p* = .018) and somatization symptoms (*β* = − 0.30, *p* = .003). In addition to shared factors, the perceived impact of COVID-19 was the unique risk factor for state anxiety (*β* = 0.34, *p* < .001).

**Table 2:** Hierarchical linear regression coefficients for the psychopathological symptoms (*N* = 119)

**Variables**	**Model of generalized anxiety**					**Model of state anxiety**					**Model of depressive symptoms**				
	Model 1	Model 2	Model 3	Model 4	Model 5	Model 1	Model 2	Model 3	Model 4	Model 5	Model 1	Model 2	Model 3	Model 4	Model 5
***Socio-demographics characteristics***															
Age	0.22*	0.01*	0.17	0.17*	0.18*	0.05	0.10	0.07	0.03	0.03	0.19*	0.21*	0.14	0.12	0.14
Sex (male)	−0.07	−0.13	−0.15	−0.17*	− 0.16	−0.06	− 0.12	− 0.11	− 0.10	− 0.09	− 0.10	− 0.13	− 0.18*	− 0.19*	− 0.17
Ethnic group (Han)	−0.05	− 0.17	− 0.04	− 0.07	− 0.08	0.07	0.03	0.01	−0.02	−0.04	−0.01	−0.03	−0.01	−0.04	−0.07
Education level	0.01	0.01	0.04	0.05	0.04	−0.04	0.00	0.11	0.13	0.12	−0.02	0.00	0.04	0.05	0.04
Annual income	−0.02	− 0.00	− 0.07	− 0.07	− 0.05	− 0.06	− 0.04	− 0.08	− 0.10	− 0.07	− 0.08	− 0.07	− 0.14	− 0.13	− 0.10
Community risk	0.23*	0.16*	0.13	0.15	0.16	0.14	0.05	0.06	0.06	0.08	0.17	0.13	0.07	0.07	0.09
Infection type	−0.03	− 0.04	−0.03	− 0.02	− 0.04	− 0.07	− 0.09	− 0.07	− 0.04	− 0.05	− 0.03	− 0.04	− 0.04	− 0.03	− 0.05
History of mental disorder (yes)	0.27**	1.19*	0.09	0.09	0.14	0.26**	0.20*	0.08	0.07	0.12	0.23*	0.21*	0.04	0.03	0.11
***COVID-19-related factors***															
Exposure to related news		0.01	0.03	0.07	0.06		0.04	0.06	0.05	0.04		0.02	0.01	0.02	0.01
Perceived impact of pandemic		0.19*	0.17*	0.15	0.16		0.45***	0.37***	0.32***	0.34***		0.20*	0.16	0.12	0.13
***Psychological factors***															
Loneliness			0.43***	0.37***	0.37***			0.33***	0.29***	0.26**			0.48***	0.44***	0.42***
Hope			0.27**	0.22*	0.23*			−0.09	−0.01	0.02			0.23*	0.24*	0.26*
***Coping strategies***															
Active coping				0.10	0.11				−0.22	− 0.22				0.02	0.03
Avoidant coping				0.21*	0.22*				0.16	0.18				0.25*	0.26**
Emotion-focused coping				0.24*	0.20				0.11	0.08				−0.03	−0.09
Acceptance coping				−0.40	− 0.43**				−0.17	−0.21				−0.15	−0.20
***Social support***															
Family support					−0.13					−0.21					−0.21
Friends support					0.006					−0.10					−0.08
Significant other support					0.21					0.32*					0.37*
*Adjusted R*^*2*^	0.10*	0.12	0.28	0.33	0.33	0.03	0.21	0.31	0.37	0.38	0.06	0.09	0.27	0.29	0.31
*△R*^*2*^		0.02	0.16***	0.06*	−0.01		0.17***	0.10***	0.06*	0.01		0.02	0.19***	0.02	0.01
**Variables**	**Model of PTSS/PTSD**					**Model of somatization**					**Model of interpersonal sensitivity**				
	Model 1	Model 2	Model 3	Model 4	Model 5	Model 1	Model 2	Model 3	Model 4	Model 5	Model 1	Model 2	Model 3	Model 4	Model 5
***Socio-demographics characteristics***															
Age	0.28**	0.29**	0.22**	0.22**	0.23**	0.14	0.14	0.10	0.10	0.10	−0.01	0.00	− 0.06	− 0.06	− 0.05
Sex (male)	−0.12	−0.14	−0.20*	−0.21*	−0.21*	−0.21*	−0.22*	−0.25**	−0.30**	−0.30**	−0.07	−0.08	−0.12	−0.12	−0.11
Ethnic group (Han)	−0.02	−0.03	0.01	−0.01	−0.02	0.13	0.12	0.14	0.11	0.10	−0.02	− 0.03	− 0.02	− 0.03	− 0.05
Education level	0.07	0.08	0.05	0.06	0.06	−0.04	−0.04	−0.01	−0.02	−0.00	−0.15	−0.14	−0.10	−0.08	−0.09
Annual income	−0.03	−0.02	−0.07	−0.06	−0.06	−0.01	−0.00	−0.05	−0.00	−0.01	−0.05	−0.05	−0.11	−0.12	−0.11
Community risk	0.19*	0.17	0.09	0.10	0.10	0.13	0.12	0.08	0.10	0.11	0.16	0.14	0.09	0.09	0.10
Infection type	0.05	0.05	0.04	0.05	0.03	0.13	0.13	0.13	0.13	0.09	0.01	0.01	0.01	0.04	0.03
History of mental disorder (yes)	0.37***	0.36***	0.23**	0.22*	0.25*	0.03	0.02	−0.09	− 0.07	− 0.05	0.47***	0.46***	0.30***	0.28**	0.31**
***COVID-19-related factors***															
Exposure to related news		0.07	0.05	0.07	0.07		0.02	0.02	0.06	0.06		0.02	0.01	0.05	0.04
Perceived impact of pandemic		0.15	0.15	0.14	0.14		0.05	0.02	0.01	−0.00		0.10	0.06	0.04	0.05
***Psychological factors***															
Loneliness			0.36***	0.34***	0.34***			0.31**	0.24*	0.23*			0.44***	0.42***	0.41***
Hope			0.33***	0.28**	0.29**			0.15	0.12	0.13			0.19*	0.12	0.13
***Coping strategies***															
Active coping				0.08	0.08				0.31*	0.28				−0.01	− 0.01
Avoidant coping				0.13	0.13				0.24*	0.25*				0.15	0.15
Emotion-focused coping				0.07	0.04				−0.01	−0.08				0.22	0.20
Acceptance coping				−0.14	− 0.15				−0.37*	−0.36				−0.18	−0.20
***Social supports***															
Family support					−0.03					0.07					−0.10
Friends support					−0.06					−0.29					−0.06
Significant other support					0.14					0.32					0.17
*Adjusted R*^*2*^	0.19***	0.20	0.34	0.34	0.32	0.05	0.03	0.10	0.14	0.15	0.21***	0.21	0.37	0.39	0.38
*△R*^*2*^		0.01	0.15***	−0.01	−0.01		−0.02	0.07**	0.04	0.01		− 0.00	0.15***	0.03	−0.01
**Variables**	**Model of hostility**					**Model of paranoid ideation**					**Model of psychoticism**				
	Model 1	Model 2	Model 3	Model 4	Model 5	Model 1	Model 2	Model 3	Model 4	Model 5	Model 1	Model 2	Model 3	Model 4	Model 5
***Socio-demographics characteristics***															
Age	0.02	0.04	−0.01	−0.01	−0.01	0.06	0.08	0.02	0.02	0.03	0.06	0.07	0.01	0.01	0.01
Sex (male)	0.00	−0.00	−0.03	− 0.03	− 0.03	− 0.00	− 0.01	−0.06	−0.08	−0.07	0.02	0.01	−0.03	−0.06	−0.05
Ethnic group (Han)	−0.10	−0.11	−0.10	−0.12	−0.12	0.03	0.01	0.04	0.03	0.02	−0.02	− 0.03	− 0.01	− 0.04	− 0.04
Education level	−0.15	−0.13	−0.11	−0.09	−0.08	−0.13	−0.11	−0.11	−0.11	−0.12	−0.13	−0.12	−0.09	−0.09	−0.11
Annual income	0.02	0.02	−0.03	−0.03	−0.03	0.02	0.02	−0.04	−0.03	−0.01	0.02	0.03	−0.03	−0.01	0.01
Community risk	0.12	0.09	0.05	0.06	0.06	0.11	0.08	0.02	0.03	0.04	0.10	0.08	0.03	0.04	0.05
Infection type	−0.01	−0.02	−0.01	0.01	−0.01	−0.00	−0.01	−0.01	−0.01	−0.02	−0.09	−0.10	−0.09	−0.09	−0.08
History of mental disorder (yes)	0.61***	0.59***	0.48***	0.47***	0.48***	0.68***	0.66***	0.53***	0.54***	0.58***	0.72***	0.71***	0.57***	0.58***	0.62***
***COVID-19-related factors***															
Exposure to related news		−0.07	−0.07	−0.04	−0.04		−0.03	−0.05	−0.02	−0.03		0.00	−0.01	0.03	0.02
Perceived impact of pandemic		0.11	0.08	0.06	0.05		0.13	0.11	0.11	0.12		0.09	0.06	0.03	0.04
***Psychological factors***															
Loneliness			0.31***	0.27***	0.27**			0.36***	0.33***	0.32***			0.37***	0.33***	0.33***
Hope			0.14	0.10	0.10			0.25**	0.21*	0.22*			0.19**	0.17*	0.18*
***Coping strategies***															
Active coping				0.00	−0.01				0.10	0.10				0.12	0.15
Avoidant coping				0.20*	0.20*				0.07	0.07				0.21**	0.22**
Emotion-focused coping				0.20	0.16				0.16	0.14				0.05	0.05
Acceptance coping				−0.25	−0.24				−0.23	−0.26				−0.25*	−0.30*
***Social supports***															
Family support					0.05					−0.14					−0.18
Friends support					−0.12					−0.01					0.17
Significant other support					0.14					0.17					0.02
*Adjusted R*^*2*^	0.37***	0.37	0.45	0.49	0.48	0.40***	0.41	0.53	0.54	0.53	0.48***	0.48	0.60	0.63	0.62
*△R*^*2*^		0.01	0.07**	0.04*	−0.01		0.01	0.12***	0.00	−0.01		−0.00	0.12***	0.03*	−0.00

*Note*. The regression coefficients in this table were standardized regression coefficients. *p < .05; **p < .01; ***p < .001

## Discussion

This study demonstrated that at least one third of hospitalized patients exhibited symptoms of depression, anxiety, and PTSS/PTSD and that the prevalence is much higher than that in the general population (approximately 20%) [36]. The reasons why higher mental health risks were observed in the patients with COVID-19 than in the general population are as follows. First, cytokines that directly or indirectly affect the brain might be induced by COVID-19, for example, it was reported that severe COVID-19 infection may cause delirium, various mental health problems, and cerebropathy [37]. Second, the social distancing measures and quarantine policies [10]. In China, patients with COVID-19 infections must be temporarily housed in isolated hospitals. These patients are more likely to have or be experiencing autism, anger, anxiety, depressive disorder, and insomnia, due to social isolation, uncertainties, and drug reactions [38, 39].

Prevalence of psychopathological symptoms among patients during the second wave of the COVID-19 pandemic in Beijing was lower than that of the first wave in Wuhan City. A preliminary survey at the end of January 2020 demonstrated that more than half of the patients diagnosed with COVID-19 infections experienced moderate to severe psychological health disorders [14]. Two reasons might explain this phenomenon. The first reason is that timely mental health care was provided: in the second wave, the health authority rapidly dispatched psychiatrists, psychiatric nurses, and clinical psychologists to support patients with COVID-19. Notably, all patients with psychological health problems should receive professional psychotherapy and appropriate mental health services. The second reason is that patients had more knowledge on COVID-19 in the second wave than in the first wave and thus an improved understanding of their physic health status. This accumulation of knowledge may help patients decrease their sense of uncertainty and fear. Additionally, our study found that prevalence of mental health symptoms was much higher when we used the self-report questionnaires than when using the MINI diagnosis. In terms of severity, total scores for depression, anxiety, and PTSS/PTSD on the self-report scales were below the clinical cutoffs, demonstrating that although most of the patients might have had psychopathological symptoms, they did not fulfill the criteria for psychopathological disorders.

Another prominent finding was that all psychopathological symptoms were associated with loneliness, which may be due to the quarantine and isolation policies and insufficient social support. This finding is similar to those in previous studies, for example, isolation can negatively affect the mental health of children and adults [40, 41]. Additionally, individuals in isolation often dislike it because they are separated from their loved ones, lose their freedoms, and are bored at home. All these factors may increase loneliness. Regarding our recommendations for mental health, officials should specify the required duration of individual isolation; convey specific reasons for the isolation and accurate information on relevant agreements; provide adequate supplies to the isolated persons; and deliver mental health interventions to the affected patients during the isolation period, for instance, psychological counseling services that use electronic devices and applications (e.g., smartphones and WeChat, respectively) as the communication media.

We also found that for some psychopathological symptoms, the avoidant coping strategy was a risk factor and the acceptance coping strategy was a protective factor. This finding indicates that patients should be encouraged to accept the reality of being infected and not to manage the infection by avoiding them. Furthermore, we found that hope was a risk factor for some symptoms. One possible explanation for this finding is that individuals with more hope have difficulty in accepting terrible situations and tend to avoid reality [42]. The results also demonstrated that support from significant others might increase patients’ state anxiety and depression. This finding is a reminder that too much unnecessary care, especially from individuals who are not family or friends but significant others (e.g., medical workers, colleagues, bosses) could increase patients’ anxiety about their illness. Thus, “moderate” care is necessary for patients with infections.

We assumed that COVID-19 might increase individual anxiety temporarily and chronically; thus, we examined two types of anxiety (i.e., state anxiety and generalized anxiety) to demonstrate temporary and chronical anxiety. Although the prevalence of generalized anxiety and state anxiety symptoms were equal, there were more risk factors for generalized anxiety than state anxiety, and social support from significant others was only a risk for state anxiety. The likely reason for this result is that generalized anxiety is a more generalized, daily-life, chronical, and constant anxiety symptom than state anxiety [43].

The sociodemographic information suggests that age and gender also influenced the mental health of patients during the second wave of the pandemic. A higher risk of generalized anxiety and PTSS/PTSD symptoms among older patients (aged 40 years and above) was observed in this study, and this finding is the opposite of that of the general population [14]. The reason for this finding may be that COVID-19 is known to exhibit a particularly severe course in individuals of advanced age and in those with accompanying chronic disease [13, 41, 44]. Therefore, older individuals exposed to COVID-19 may be more severely affected than their younger counterparts, and older patients with infections might be more susceptible to some mental disorders, such as generalized anxiety and PTSS/PTSD than younger patients. Being female was an effective predictor of PTSS/PTSD symptoms among infected patients [45]. Our results demonstrated that PTSS/PTSD and somatization symptoms were observed more often in females than in males; this finding is consistent with those of other studies in previous studies [45]. We also found that COVID-19-related factors were not the predominant factors associated with psychopathological symptoms. For instance, exposure to COVID-19-related information or news had no significant relation across all psychopathological symptoms, indicating that psychopathological symptoms are related to more stable variables, such as a history of mental disorders.

This study has several limitations. First, the sample size was small because of the limited number of positive cases (i.e., *n* = 331) during the second wave in Beijing. Second, due to the absence of a baseline psychiatric assessment, the incidence rate estimate was inaccurate and had to rely on the point prevalence rate when conditions permitted. Third, the reliability of the exposure to COVID-19-related information and loneliness was lower than 0.6 and thus greater caution was necessary in making inferences. Finally, no objective biological indicators were included. In further research, other indicators such as peripheral blood heredity, inflammation, immune and metabolic function markers, cerebrospinal fluid indicators, electroencephalogram (EEG), or brain imaging are necessary.

## Conclusions

In summary, this study investigated the prevalence of psychopathological symptoms and related risk factors among hospitalized patients with COVID-19 infection during the second wave of the pandemic. Clinicians must be aware that hospitalized patients may experience higher rates and severity of depressive symptoms, anxiety symptoms, posttraumatic stress disorder, and other neuropsychiatric syndromes than the general population. Therefore, we recommend that the Chinese government act with urgency to provide patients with COVID-19 infections the specific mental health interventions and resources that will help them manage the virus and solve corresponding mental health problems.

## Data Availability

The datasets used during the current study are available from the corresponding author on reasonable request.

## References

[1] Huang C, Wang Y, Li X, Ren L, Zhao J, Hu Y (2020). Clinical features of patients infected with 2019 novel coronavirus in Wuhan, China. Lancet.

[2] Wang C, Horby PW, Hayden FG, Gao GF (2020). A novel coronavirus outbreak of global health concern. Lancet.

[3] Xu S, Li Y (2020). Beware of the second wave of COVID-19. Lancet.

[4] Yuanyuan W, Zhishan H, Yi F, Amanda W, Runsen C (2020). Changes in network centrality of psychopathology symptoms between the COVID-19 outbreak and after peak. Mol Psychiatry.

[5] Wu JT, Leung K, Leung GM (2020). Nowcasting and forecasting the potential domestic and international spread of the 2019-nCoV outbreak originating in Wuhan, China: a modelling study. Lancet.

[6] Troyer EA, Kohn JN, Hong S (2020). Are we facing a crashing wave of neuropsychiatric sequelae of COVID-19? Neuropsychiatric symptoms and potential immunologic mechanisms. Brain Behav Immun.

[7] Candel FJ, Barreiro P, San Román J, Abanades JC, Barba R, Barberán J, Bibiano C, Canora J, Cantón R, Calvo C, Carretero M, Cava F, Delgado R, García-Rodríguez J, González del Castillo J, González de Villaumbrosia C, Hernández M, Losa JE, Martínez-Peromingo FJ, Molero JM, Muñoz P, Onecha E, Onoda M, Rodríguez J, Sánchez-Celaya M, Serra JA, Zapatero A, Servicio de Enfermedades Infecciosas y Microbiología Clínica. Hospital Universitario San Carlos. Madrid. Spain (2020). Recommendations for use of antigenic tests in the diagnosis of acute SARS-CoV-2 infection in the second pandemic wave: attitude in different clinical settings. Revista espanola de quimioterapia : publicacion oficial de la Sociedad Espanola de Quimioterapia.

[8] Vindegaard N, Benros ME (2020). COVID-19 pandemic and mental health consequences: systematic review of the current evidence. Brain Behav Immun.

[9] Lewnard JA, Lo NC (2020). Scientific and ethical basis for social-distancing interventions against COVID-19. Lancet Infect Dis.

[10] Brooks SK, Webster RK, Smith LE, Woodland L, Wessely S, Greenberg N (2020). The psychological impact of quarantine and how to reduce it: rapid review of the evidence. Lancet.

[11] Asmundson GJG, Taylor S (2020). Coronaphobia: fear and the 2019-nCoV outbreak. J Anxiety Disord.

[12] Xiang YT, Yang Y, Li W, Zhang L, Zhang Q, Cheung T, Ng CH (2020). Timely mental health care for the 2019 novel coronavirus outbreak is urgently needed. Lancet Psychiatry.

[13] Zhang J, Lu H, Zeng H, Zhang S, Du Q, Jiang T (2020). The differential psychological distress of populations affected by the COVID-19 pandemic. Brain Behav Immun.

[14] Shi L, Lu ZA, Que JY, Huang XL, Liu L, Ran MS, Gong YM, Yuan K, Yan W, Sun YK, Shi J, Bao YP, Lu L (2020). Prevalence of and risk factors associated with mental health symptoms among the general population in China during the coronavirus disease 2019 pandemic. JAMA Netw Open.

[15] Özdin S, Bayrak ÖŞ (2020). Levels and predictors of anxiety, depression and health anxiety during COVID-19 pandemic in Turkish society: the importance of gender. Int J Soc Psychiatry.

[16] Feng Y, Zong M, Yang Z, Gu W, Dong D, Qiao Z (2020). When altruists cannot help: the influence of altruism on the mental health of university students during the COVID-19 pandemic. Glob Health.

[17] Gierveld JDJ, Tilburg TV (2006). A 6-item scale for overall, emotional, and social loneliness: confirmatory tests on survey data. Res Aging.

[18] Snyder CR (1995). Conceptualizing, measuring, and nurturing hope. J Counseling Dev.

[19] Phelps SB, Jarvis PA (1994). Coping in adolescence: empirical evidence for a theoretically based approach to assessing coping. J Youth Adolescence.

[20] Carver CS, Scheier MF, Weintraub JK (1989). Assessing coping strategies: a theoretically based approach. J Pers Soc Psychol.

[21] Zimet GD, Dahlem NW, Zimet SG, Farley GK (1988). The multidimensional scale of perceived social support. J Pers Assess.

[22] Spitzer RL, Kroenke K, Williams JBW, Lowe B (2006). A brief measure for assessing generalized anxiety disorder: the GAD-7. Arch Intern Med.

[23] Tong X, An D, McGonigal A, Park S-P, Zhou D (2016). Validation of the generalized anxiety Disorder-7 (GAD-7) among Chinese people with epilepsy. Epilepsy Res.

[24] Okun A, Stein RE, Bauman LJ, Silver EJ (1996). Content validity of the psychiatric symptom index, CES-depression scale, and state-trait anxiety inventory from the perspective of DSM-IV. Psychol Rep.

[25] Teichman Y, Melinick C (1979). Manual for the state-trait anxiety inventory (STAI). Israel: Tel-Aviv University.

[26] Dennis C-L, Coghlan M, Vigod S (2013). Can we identify mothers at-risk for postpartum anxiety in the immediate postpartum period using the state-trait anxiety inventory?. J Affect Disord.

[27] Kroenke K, Spitzer RL (2002). The PHQ-9: a new depression diagnostic and severity measure. Psychiatr Ann.

[28] Wang W, Bian Q, Zhao Y, Li X, Wang W, Du J (2014). Reliability and validity of the Chinese version of the patient health questionnaire (PHQ-9) in the general population. Gen Hosp Psychiatry.

[29] Weiss DS (2007). The impact of event scale: revised. Cross-cultural assessment of psychological trauma and PTSD.

[30] Wang Y, Duan Z, Ma Z, Mao Y, Li X, Wilson A (2020). Epidemiology of mental health problems among patients with cancer during COVID-19 pandemic. Transl Psychiatry.

[31] Tan W, Hao F, McIntyre RS, Jiang L, Jiang X, Zhang L (2020). Is returning to work during the COVID-19 pandemic stressful? A study on immediate mental health status and psychoneuroimmunity prevention measures of Chinese workforce. Brain Behav Immun.

[32] Lia X, Lib S, Xianga M, Fanga Y, Qiana K, Xua J (2020). The prevalence and risk factors of PTSD symptoms among medical assistance workers during the COVID-19 pandemic. J Psychosom Res.

[33] Boulet J, Boss MW (1991). Reliability and validity of the brief symptom inventory. Psychol Assess J Consulting Clin Psychol.

[34] Ruckenstein MJ, Staab JP (2001). The basic symptom inventory—53 and its use in the Management of Patients with psychogenic dizziness. Otolaryngol Head Neck Surg.

[35] Cohen J, Cohen P, West SG, Aiken LS (2013). Applied multiple regression/correlation analysis for the behavioral sciences.

[36] Wang C, Pan R, Wan X, Tan Y, Xu L, McIntyre RS (2020). A longitudinal study on the mental health of general population during the COVID-19 epidemic in China. Brain Behav Immun.

[37] Andrews LJ, Benken ST (2020). COVID-19: ICU delirium management during SARS-CoV-2 pandemic-pharmacological considerations. Critical care.

[38] Bo HX, Li W, Yang Y, Wang Y, Zhang Q, Cheung T, et al. Posttraumatic stress symptoms and attitude toward crisis mental health services among clinically stable patients with COVID-19 in China. Psychol Med. 2020:1–2. 10.1017/S0033291720000999. 10.1017/S0033291720000999 PMC7200846 32216863

[39] Lin CY, Peng YC, Wu YH, Chang J, Chan CH, Yang DY (2007). The psychological effect of severe acute respiratory syndrome on emergency department staff. Emerg Med J.

[40] Zhou J, Liu L, Xue P, Yang X, Tang X (2020). Mental health response to the COVID-19 outbreak in China. Am J Psychiatry.

[41] Fernández-Aranda F, Casas M, Claes L, Bryan DC, Favaro A, Granero R, Gudiol C, Jiménez-Murcia S, Karwautz A, le Grange D, Menchón JM, Tchanturia K, Treasure J (2020). COVID-19 and implications for eating disorders. Eur Eat Disord Rev.

[42] Snyder CR (2011). Conceptualizing, measuring, and nurturing Hope. J Counseling Dev..

[43] Crocq MA (2017). The history of generalized anxiety disorder as a diagnostic category. Dialogues Clin Neurosci.

[44] Zhou F, Yu T, Du R, Fan G, Liu Y, Liu Z (2020). Clinical course and risk factors for mortality of adult inpatients with COVID-19 in Wuhan, China: a retrospective cohort study. Lancet.

[45] Liu N, Zhang F, Wei C, Jia Y, Shang Z, Sun L, Wu L, Sun Z, Zhou Y, Wang Y, Liu W (2020). Prevalence and predictors of PTSS during COVID-19 outbreak in China hardest-hit areas: gender differences matter. Psychiatry Res.

